# Alleviation of pulmonary fibrosis by the dual PPAR agonist saroglitazar and breast milk mesenchymal stem cells via modulating TGFß/SMAD pathway

**DOI:** 10.1007/s00210-024-03004-y

**Published:** 2024-02-20

**Authors:** Seba Hassan Attia, Sara F. Saadawy, Samaa M. El-Mahroky, Mahitab M. Nageeb

**Affiliations:** 1 Clinical Pharmacology Department, Faculty of Medicine, Zagazig University, Zagazig, Egypt; 2 Medical Biochemistry and Molecular Biology Department, Faculty of Medicine, Zagazig University, Zagazig, Egypt; 3 Medical Histology and Cell Biology Department, Faculty of Medicine, Zagazig University, Zagazig, Egypt

**Keywords:** Lung fibrosis, PPAR, Saroglitazar, Stem cells, Autophagy

## Abstract

Pulmonary fibrosis (PF) is a complex disorder with high morbidity and mortality. Limited efficacies of the available drugs drive researchers to seek for new therapies. Saroglitazar (Saro), a full (PPAR α/γ) agonist, is devoid of known PPAR-mediated adverse effects. Breast milk mesenchymal stem cells (BrMSCs) are contemplated to be the ideal cell type harboring differentiation/anti-inflammatory/immunosuppressive properties. Accordingly, our aims were to investigate the potential roles of Saro and/or BrMSCs in PF and to spot their underlying protective mechanisms. In this study, PF was induced by bleomycin (BLM) via intratracheal instillation. Treatment started 14 days later. Animals were treated with oral saroglitazar (3 mg/kg daily) or intraperitoneal single BrMSCs injection (0.5 ml phosphate buffer saline (PBS) containing 2 × 10^7^ cells) or their combination with same previous doses. At the work end, 24 h following the 6 weeks of treatment period, the levels of oxidative (MDA, SOD), inflammatory (IL-1ß, IL-10), and profibrotic markers (TGF-ß, αSMA) were assessed. The autophagy-related genes (LC3, Beclin) and the expression of PPAR-α/γ and SMAD-3/7 were evaluated. Furthermore, immunohistochemical and histological work were evaluated. Our study revealed marked lung injury influenced by BLM with severe oxidative/inflammatory/fibrotic damage, autophagy inhibition, and deteriorated lung histology. Saro and BrMSCs repaired the lung structure worsened by BLM. Treatments greatly declined the oxidative/inflammatory markers. The pro-fibrotic TGF-ß, αSMA, and SMAD-3 were decreased. Contrarily, autophagy markers were increased. SMAD-7 and PPAR α/γ were activated denoting their pivotal antifibrotic roles. Co-administration of Saro and BrMSCs revealed the top results. Our findings support the study hypothesis that Saro and BrMSCs can be proposed as potential treatments for IPF.

**Supplementary Information:**

The online version contains supplementary material available at 10.1007/s00210-024-03004-y.

## Introduction

Pulmonary fibrosis (PF), a devastating fibro-proliferative disease, is developed secondary to various lung disorders (Zakaria et al. 2021). However, cigarette smoking and chronic exposure to mineral dust are the most important triggering factors of disease progression (Li et al. 2017). Lung scarring following severe coronavirus disease (COVID-19) infection is an added causative factor of PF (Hama Amin et al. 2022). Among all causes, the most threatening form is idiopathic pulmonary fibrosis (IPF) which has an unidentified etiology (Johannsonet al. 2018; Bellou et al. 2021).

Despite the mysterious pathogenesis of PF, inflammatory cells infiltration, intense oxidative injury, excessive proliferation of fibroblasts, and their transformation to myofibroblasts, massive accumulation of extracellular matrix (ECM) proteins and collagen deposition into the lung parenchyma are the disease main characteristic factors (Mansouri et al. 2019).

Interleukins and transforming growth factor-β (TGF-β) are the principal driving inflammatory cytokines of PF. They are found to be highly expressed in the damaged lungs promoting ECM deposition and hastening the pro-fibrotic response (Walton et al. 2017). Notably, induction of intracellular signaling by TGF-β1 ligands activated the receptor-regulated proteins (SMAD-2 and 3) which regulate the expression of the pro-fibrotic genes (Walton et al. 2017; Yamazaki et al. 2017). In contrary, SMAD-7 wields opposing activities via braking TGF-β1 induced expression of pro-fibrotic genes by blocking the phosphorylation of SMAD-2/3 (Lv et al. 2019). The anti-fibrotic effect of SMAD-7 significantly demonstrated protective actions against TGF-β1 mediated PF (Qin et al. 2019; Li et al. 2021).

Autophagy, the crucial cellular mechanism activated by multiple intracellular or extracellular aspects, is linked to plenty of genes among which Beclin1 and microtubule-associated proteins 1A/1B light chain 3 (LC-3) are essential regulators (Levine and Kroemer 2008; Liu et al. 2022; Nieto-Torres et al. 2021). In IPF, suppression of oxidative stress stimulates autophagy and therefore the process of fibrogenesis is alleviated (Zhang et al. 2021). Various studies addressed that autophagy is blocked in the lungs of IPF patients (Araya et al. 2013; Patel et al. 2012).

Peroxisome proliferator-activated receptors (PPARs) are widely expressed nuclear receptors which dynamically regulate cell functions in a versatile manner via controlling the transcription of genes involved in lipid and carbohydrate metabolism (Kumar et al. 2020). PPAR-γ modulates the expression of genes linked not only to glucose homeostasis (Picard and Auwerx 2022), inflammatory responses (Clark 2002), apoptosis (Chang and Szabo 2000), but also plays a central role in regulating fibrogenesis mechanisms in many tissues including the lung (Yoon et al. 2015). The anti-fibrotic action of PPAR-γ is displayed via interfering with TGF-ß signaling pathways either being SMAD dependent or independent (Yoon et al. 2015). In the meanwhile, PPAR-α activation showed a fundamental role in the recovery of lung function after acute lung injury via exhibiting an anti-inflammatory action (Liu et al. 2017a, b). On that account, drugs targeting PPARs have an interesting potential in tackling inflammatory and fibrogenic mechanisms. Saroglitazar the mixed PPAR α/γ agonist is an approved safe drug for diabetic dyslipidemia and hypertriglyceridemia (Gawrieh et al. 2021). Literatures reported its beneficial role in mitigating hepatic fibrosis and non-alcoholic steatohepatitis via repressing TGF-β1 and modulating the inflammatory cytokines (Makled et al. 2019; Lange et al. 2022; Akbari et al. 2021).

Stem cells, the cornerstone of regenerative medicine, are one of the excellent paradigms used in the management of many diseases because of their unique abilities to differentiate into various cell lines and their self-renewal capacity (Khamis et al. 2021). The adult mesenchymal stem cells (MSCs) could be isolated from many sources including breast milk which is a highly reproducible and non-invasive source (Nageeb et al. 2022). Transplantation of MSCs from many sources proved their ability to repair damaged lung tissue in PF; however, up to our knowledge, the role of breast milk mesenchymal stem cells (BrMSCs) on PF was not investigated yet (Chu et al. 2020; Kletukhina et al. 2022). It is worth noting that, BrMSCs have demonstrated their beneficial roles on various disorders (Khamis et al. 2021; Nageeb et al. 2022; Hamid et al. 2022).

Despite the presence of different therapeutics that beat the life-threatening PF, the unavoidable adverse effects, decreased tolerability, limited efficacy, and unsatisfactory prognosis of the disease make them not truly beneficial (Vacchi et al. 2020). Meanwhile, considering the well-recognized favorable roles of PPAR activation in ameliorating tissue fibrosis, we hypothesized that activation of PPARs by saroglitazar may provide an excellent control of the causative PF pathways. We also aimed to compare saroglitazar with BrMSCs as another treatment option, particularly with the interesting safety profiles of both therapies.

## Materials and methods

### Animals and drugs

The experimentation was conducted on fifty Wistar male rats in the animal house at the Faculty of Medicine, Zagazig University, Egypt. Rats weighting 200 ± 20 g (age, 6–8 weeks) were purchased from the Faculty of Veterinary Medicine, Zagazig University, Egypt. Animals were housed for one week acclimatization period under precise pathogen-free conditions with standard humidity, 12 h light/dark cycles, and a temperature of 22 ± 2°C. Standard pelleted food and water were given ad libitum to rats.

Bleomycin sulfate was commercially purchased from Sigma/Aldrich Chemical Co. (Merck Life Science, Gillingham, UK). Saroglitazar was purchased from Zydus Cadila (Ahmedabad, India). Unless otherwise noted, all other chemicals of analytical grade used in this study were obtained from Sigma/Aldrich.

### Animals grouping and induction of pulmonary fibrosis (PF)

**Fig. 1:**
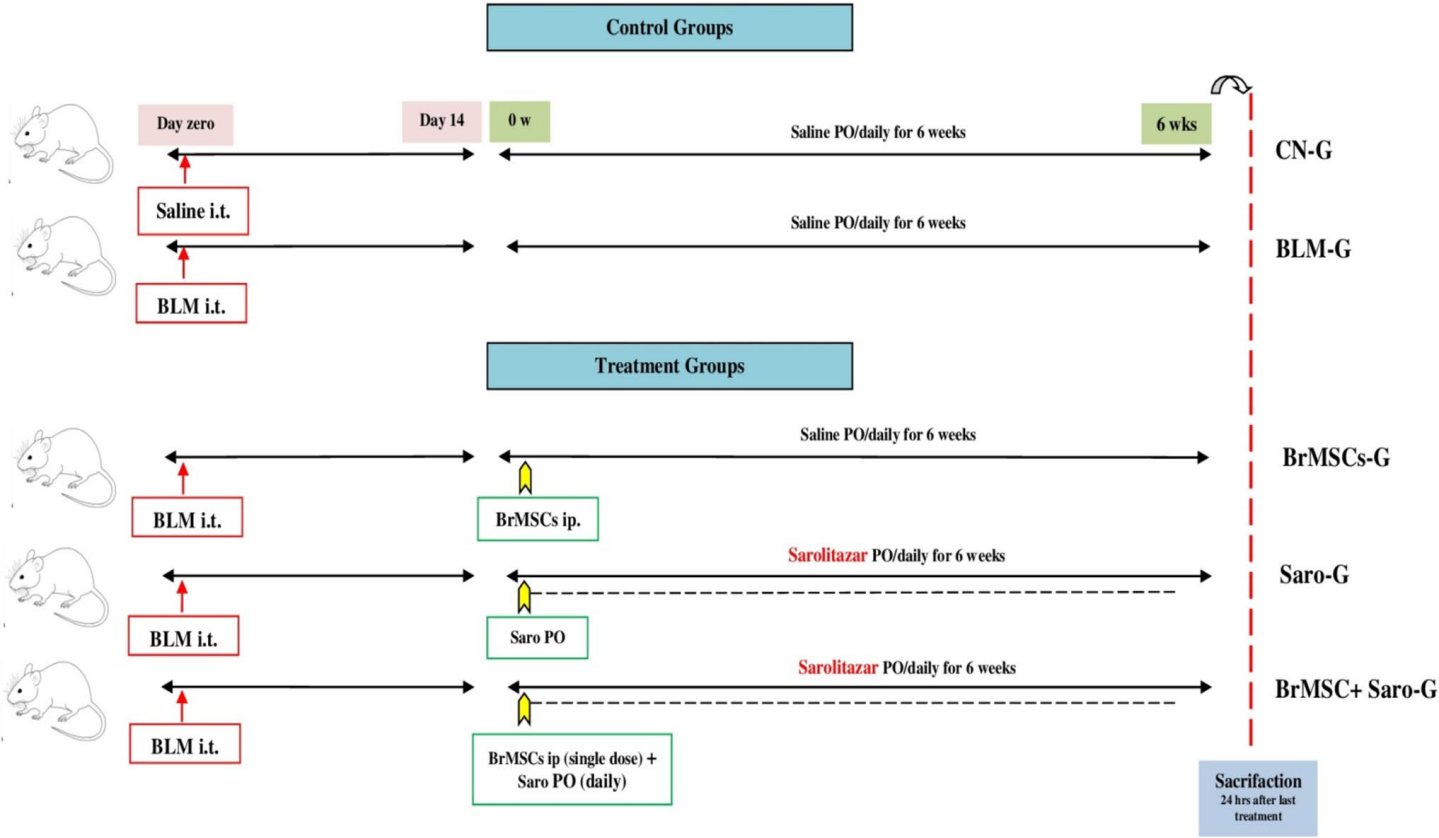
Illustrated diagram of the experimental design

As seen in the diagram illustrating the experimental design (Fig. 1), the experimental rats were randomly assigned into two major groups as follows: Control and treated groups. Control rats (*n* = 20) were subdivided equally into control normal (CN-G) and control model (BLM-G). In the latter group, lung fibrosis was induced via non-surgical instillation of bleomycin (BLM) through intratracheal (i.t.) injection of a single bleomycin dose of (5 mg/kg) dissolved in 0.1 ml normal saline (Zakaria et al. 2021). The i.t. BLM instillation was done once on day 0 for induction of PF, and treatments started 14 days post-induction.

In brief, rats were intraperitoneally injected with ketamine/xylazine anesthesia. With assistance, the animal was stabilized in the upright position while its neck was pulled backward. To visualize the trachea, a blunt forceps was used to pull out the rats’ tongue gently to one side. Afterward, bleomycin was intratracheally instilled using a syringe fitted into a bent gavage needle and smoothly pushed against the soft palate to reach the trachea. Normal control rats received the same volume of saline (10 ml/kg body weight) via i.t. injection once, then after 14 days, the control rats were daily administered saline orally for six weeks **(**Mehdizadeh et al. 2022).

Treatment started 14 days after BLM i.t. injection following modeling of PF (Zakaria et al. 2021). In the treated groups (*n* = 30 rats), where lung fibrosis was also induced, the animals were subdivided equally into the following:

- Breast milk mesenchymal stem cells treated group (BrMSCs-G) where rats intraperitoneally injected with 0.5 ml phosphate buffer saline (PBS) containing 2 × 10^7^ cells of BrMSCs in a single dose (Nageeb et al. 2022).
- Saroglitazar treated group (Saro-G) where rats received oral saroglitazar 3 mg/kg daily for six weeks (Makled et al. 2019).
- Combined group (BrMSCs + Saro-G) where rats received both BrMSCs and saroglitazar in the same previous doses. In the combination group, the intraperitoneal injection of BrMSCs was 6 h away from the orally administered saroglitazar (Saro), and then Saro was received at the same time each day for 6 weeks.

Twenty-four hours after the last dose of treatment, eight weeks after BLM i.t. instillation, sacrifaction was done for estimation of all parameters after harvesting the lungs and blood.

### Collection, preparation, identification, and culturing of Br-MSCs

#### Collection, preparation, and culturing of Br-MSCs

From the nursing women and under complete aseptic circumstances, the milk samples were collected. The preparation and culturing procedures were performed according to Patki et al. (2010) protocol. Concisely, 1:2 dilution of breast milk with high glucose DMEM (Lonza Bioproducts Walkersville, MD 21793–0127 USA) was done with a mixture of penicillin–streptomycin-amphotericin B as 10 IU/10 IU/25 mg. Afterward, the breast milk was centrifuged for 10 min at 285 g. Eventually, the cell pellet in 25 cm^2^ tissue culture flasks made by Lonza Bioproducts in Walkersville, (MD 21793–0127 USA) including 10% fetal bovine serum (FBS), penicillin–streptomycin, and amphotericin B was planted.

Incubation of the flasks in a CO_2_ incubator (Heraeus, Hanau, Germany) at 37°C was done with 5% CO_2_ and 95% relative humidity. Then, as the primary passage, changing the medium was accomplished every 48 h for 14 days. Cells were trypsinized with 0.25% trypsin containing 0.02% EDTA (Lonza Bioproducts) for 5 min at 37°C and then centrifuged at (2400 rpm for 20 min) once they had reached 80% confluency. Cells were counted by using a hemocytometer, and their viability was determined using a trypan blue stain. Before the transplant, the sterilized cell pellet was re-suspended in DMEM.

#### Characters and homing of BrMSCs

**Fig. 2:**
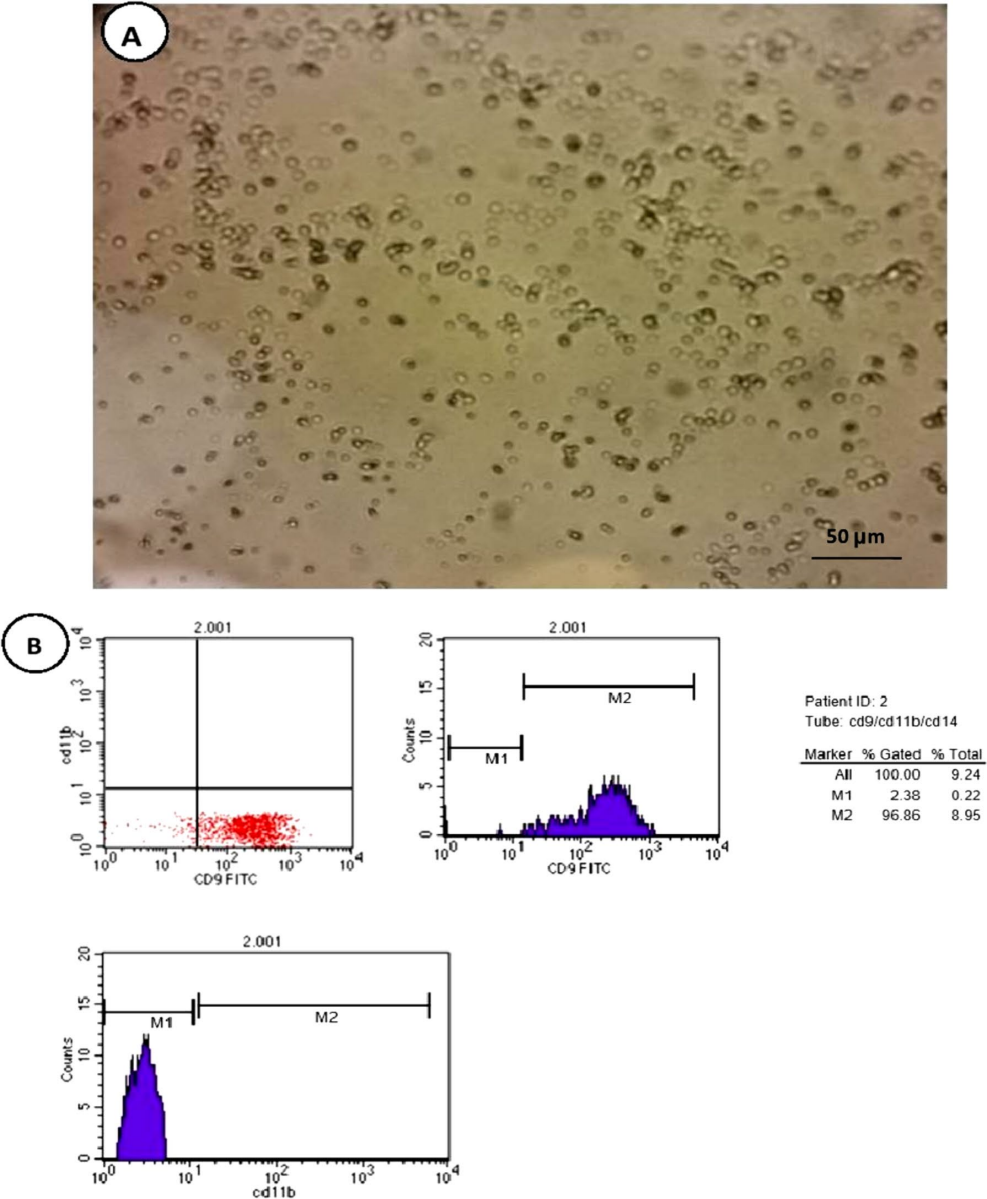
Isolation, identification, and detection of BrMSCs in lung tissue of rats. **A** BrMSCs isolation on the 3rd day of culture. **B** BrMSCs cell population were negative to hematopoietic stem cell surface marker CD 11b and positive expression of MSCs surface marker CD 9. (Magnification X 400) (scale bar = 50 μm)

Mesenchymal stem cells (MSCs) in culture were recognized by their adhesiveness and characteristic fusiform shape under the inverted microscope. Gene expression analysis for lung tissue demonstrated positive expression of MSCs surface marker (human CD9) (1.09 ± 0.25) and negative expression of hematopoietic markers (human CD 11b) in the MSCs treated groups which were confirmed by flow cytometry (Fig. 2).

### Sampling and tissue dissection

At the study end, 24 h after the last treatment, rats’ body weights were estimated. Thereafter animals were sacrificed by decapitation for the collection of blood and tissue samples after being anesthetized by isofluorane inhalation. Soon blood was collected from the retro-orbital venous plexus. Separation of sera was done for 10 min by centrifugation at 3000 rpm. Lungs were harvested, rinsed with saline, and weighed for calculation of lung index as follows: lung weight (g) /body weight ratio (g) multiplied by 100 (Turgut et al. 2016).

Preparation of lung homogenate was done from the isolated right lobes from all lungs; however, the isolated left lobes were kept in formalin 10% for histopathological examination.

### Assessment of lung lipid peroxidation and antioxidant enzyme

The thiobarbituric acid method was used to calculate the amount of malondialdehyde (MDA) in the lung tissue homogenate (Liu et al. 1997). As directed by the manufacturer, a biodiagnostic kit with the catalog number MD 2529 was utilized. However, the superoxide dismutase enzyme (SOD) antioxidant activity was assessed using the approach described by Marklund and Marklund (Marklund and Marklund 1974). Using a biodiagnostic kit (catalog no. SD 2521), the difference between the color’s absorbance at 430 nm at 0 min and after 10 min was recorded to determine the enzyme activity.

### Enzyme-linked immunosorbent assay (ELISA) measurements in lung tissue homogenate

Rat-specific sandwich enzyme-linked immunosorbent assay kits were used to assess the inflammatory (Interleukin-1 beta) and the anti-inflammatory (Interleukin-10) cytokines in lung tissue homogenate. Interleukin 1β ELISA Kits (My BioSource, CA, USA) (Cat no: MBS825017) and Interleukin-10 ELISA kits (My BioSource, CA, USA) (Cat no: MBS355232) analysis was performed according to the manufacturer’s instructions.

Lung content of transforming growth factor-beta (TFG-β) was measured using the following rat TGF-β ELISA Kit (My BioSource, CA, USA) (Cat no: MBS824788) according to manufacturer’s instructions.

Also, a rat-specific ELISA kit was used to assess the autophagy related protein, Beclin-1, (My BioSource, CA, USA) (Cat no: MBS901662). Analysis was performed according to the manufacturer’s instructions.

### Quantitative polymerase chain reaction (qPCR) analysis for the gene expression studies

With using Trizol reagent (Thermo Fisher Scientific; Waltham, MA, United States), total RNA was isolated from 30 mg of rat lung tissue. The content and purity of the extracted RNA were assessed using the NanoDrop spectrophotometer by measuring the OD at 260 and 280 nm and accepting A260/A280 at a ratio of 1.8–2.1 followed by a two-step real-time PCR to evaluate gene expression as reported previously Arisha et al., (Arisha et al. 2019). In brief, Hi Sen Script™RH (-) cDNA Synthesis Kit (iNtRON Biotechnology Co., South Korea) was used to create the cDNA. The relative gene expression was performed in a 20-μl reaction mixture having 50 ng cDNA, 1 μl of 10 pmol of each primer (forward and reverse) illustrated in the supplementary table (S1), and 10 μl qPCR 2X PreMIX (SYBR Green with low ROX) (Cat. # P725 or P750) using (Stratagene Mx3005P-qPCR System) and GAPDH was used as an internal standard control.

The cycling conditions were as follows: 95°C for 12 min first for enzyme activation, then cycling for 40 cycles at 95°C for 30 s, 60°C for 60 s, and 70°C for 60 s. Target gene expression was calculated using the 2^−ΔΔCT^ comparative method (Livak and Schmittgen 2001).

### Histopathological examination

The thoracic cages of all animals were opened, the lungs were dissected and samples from the left lobes of the lungs were taken for light microscope study. Specimens were fixed in 10% buffered formalin and managed to prepare 5-μm-thick paraffin sections for different stains: hematoxylin and eosin (H&E) stain and Mallory’s trichrome stain (Bancroft and Lyaton. 2019).

### Immunohistochemical analysis for alpha-smooth muscle actin (α-SMA) and transforming growth factor-β1 (TGFβ -1)

For immunohistochemical studies, paraffin Sects. (4 μm) were dewaxed, hydrated, and microwave-treated, and then blocked in a normal mouse serum. The sections were incubated with the specific primary antibody overnight (4°C) then the sections were incubated with the appropriate secondary antibody; a biotinylated anti-mouse IgG incubation of sections in horseradish peroxidase-avidin biotin complex (Vectastain Elite, Vector, CA) for 30 min at room temperature was done then 3,3`-diaminobenzidine in H2 O2 (DAB kit, Vector, CA) was added to visualize the reaction as a brown colored product. Sections were then counterstained with hematoxylin and mounted. To localize alpha-smooth muscle actin (α-SMA) protein as a marker for myofibroblasts, anti-alpha-smooth muscle actin antibody (rabbit polyclonal antibody; No. ab5694; dilution 1/50; Abcam, Cambridge, UK) (Egger et al. 2013) and with a monoclonal rabbit anti-TGFβ1 antibody (Clone TB21, MCA797, Serotec, Oxford, UK) at 1:200 dilution for TGF-β1 detection (Attia et al. 2017).

### Morphometric study

Leica Qwin 500 Image Analyzer Computer System (Leica Ltd, Cambridge, UK) in the unit of image analysis, Pathology Department, Faculty of Dentistry, Cairo University was used to measure the mean area percentage of collagen fibers in Mallory's trichrome stained sections (X 400), TGF-β1 and α-SMA positive reaction (X 400). The measuring frame of a standard area was 7286.78 µm^2^. Ten different nonoverlapping fields from each slide were assessed for each parameter.

### Statistical analysis

Data were expressed as mean ± standard deviation (SD) by using SPSS software (Version 22.0) (SPSS Inc, Chicago, USA). Results were accepted to be statistically significant when the *p*-value is < 0.05. One-way analysis of variances (one-way ANOVA) followed by post hoc least significant difference (LSD) for multiple comparisons was used to estimate the statistical difference between the studied groups. Pearson’s correlation coefficient was calculated to estimate the linear relationship between certain parameters.

## Results

### Effect of BrMSCs and/or saroglitazar on rats’ body weight (BW) and lung index

Bleomycin administration resulted in a marked significant (*p* < 0.05) reduction in BW compared to control normal group (CN-G). In contrast, saroglitazar and/or BrMSCs resulted in significant (*p* < 0.05) elevation of rats’ BW when compared to the bleomycin group (BLM-G). In between treated groups, the highest BW was observed in Saro-G; however, it was non-significant compared to BrMSCs-G and BrMSCs-G + Saro-G (Table 1).

**Table 1:** Effect of BrMSCs and/or saroglitazar on body weight and lung index of rats with bleomycin-induced lung fibrosis

Groups	CN-G	BLM-G	BrMSCs-G	Saro-G	BrMSCs + Saro-G
Body weight	305 ± 10^a^	256 ± 11^b^	277 ± 7.5^c^	285 ± 8.95^c^	281 ± 9.9^c^
Lung index LW(g)/BW(g) (× 100)	1.38 ± 0.08^a^	1.94 ± 0.06^b^	1.57 ± 0.05^c^	1.45 ± 0.09^ cd^	1.52 ± 0.07^c^

Values with the same superscript letters have no statistical significance difference

Values were expressed as (mean ± SD). One-way ANOVA was used for statistical analysis

*CN-G*, control normal group; *BLM-G*, lung fibrotic model group induced by bleomycin; *BrMSCs-G*, breast milk mesenchymal stem cells treated group; *Saro-G*, saroglitazar treated group; *BrMSCs* + *Saro-G*, breast milk mesenchymal stem cells and saroglitazar treated group

^a^(*p* < 0.05) when comparing CN-G with other groups

^b^(*p* < 0.05) when comparing BLM-G with other groups

^c^(*p* < 0.05) when comparing BrMSCs + Saro-G with other groups

^d^(*p* < 0.05) when comparing Saro-G with BrMSCs-G

Lung index was estimated in all studied groups as a parameter for lung edema evaluation. In contrast with body weight loss, animals of the BLM-G showed a significant (*p* < 0.05) increase in lung index as compared to CN-G. On the other hand, saroglitazar and/or BrMSCs administration demonstrated significantly (*p* < 0.05) decreased lung index in respect to BLM-G. In between treated groups, Saro-G showed the lowest lung index that was significant (*p* < 0.05) compared to BrMSCs-G and non-significant compared to the combination group (Table 1).

### Effect of BrMSCs and/or saroglitazar on oxidative stress markers of BLM-injected rats’ lung homogenates

**Fig. 3:**
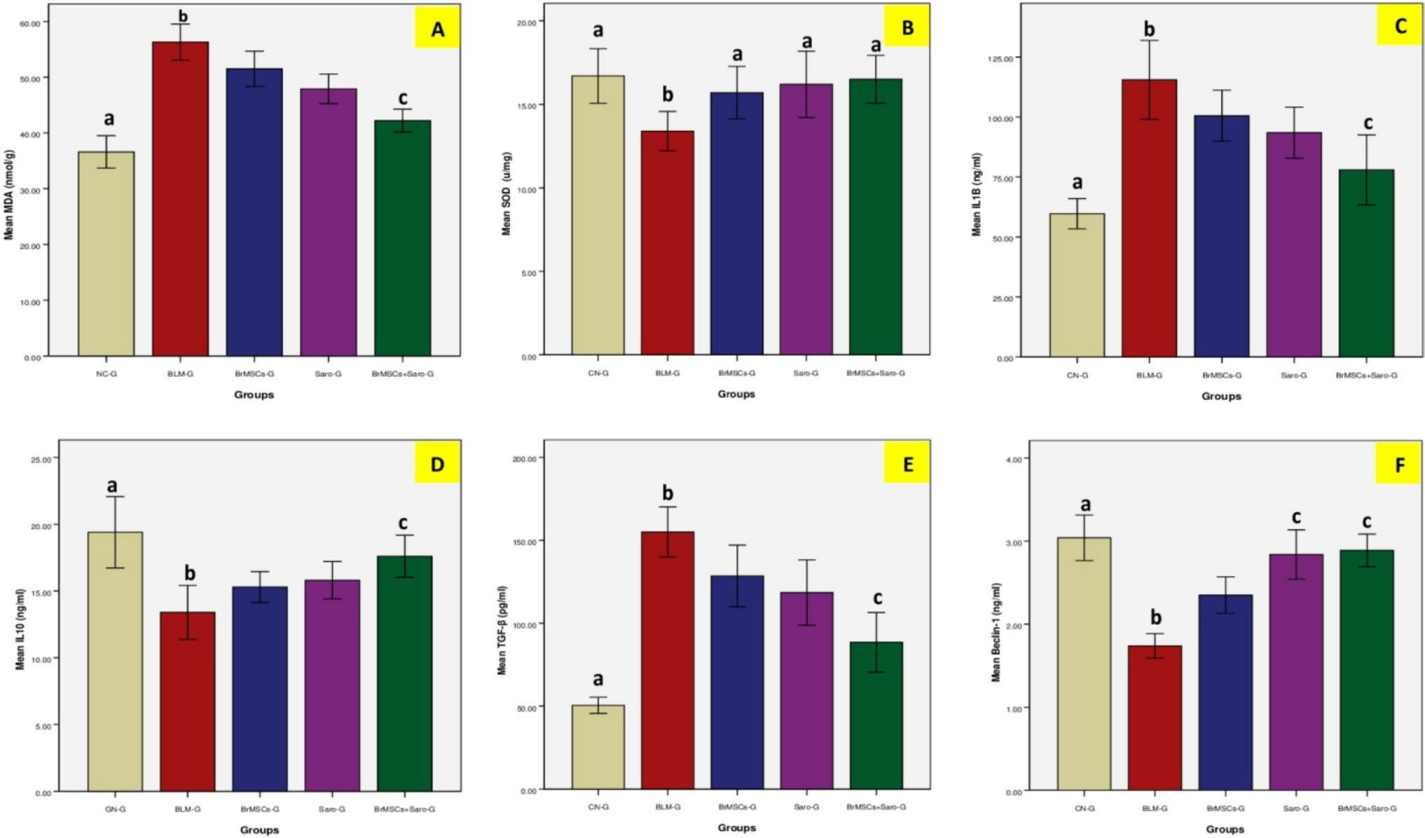
Effect of BrMSCs and/or saroglitazar on lipid peroxidation parameters **A** MDA and **B** SOD, the pro-inflammatory cytokines **C** IL-1β, **D** IL-10, **E** TGF-ß, and **F** serum Beclin-1 in rats with BLM-induced lung fibrosis

The primary contributor to BLM-induced lung fibrosis is the imbalance between the production of reactive oxygen species (ROS) and the ability of the lung tissue to detoxify these reactive products. The present study showed that i.t. injection of BLM was associated with significantly (*p* < 0.05) higher tissue levels of malondialdehyde (MDA) compared to CN-G. However, rats treated 14 days after pulmonary fibrosis induction with saroglitazar and/or BrMSCs revealed significant (*p* < 0.05) lower levels of MDA in comparison to BLM-G with remarkably superior results in the combination group (BrMSCs + Saro-G). Concerning superoxide dismutase (SOD), it was found that its enzymatic activity revealed significantly (*p* < 0.05) lower values after BLM administration in comparison to CN-G. Despite that, treatment with saroglitazar and/or BrMSCs that was initiated 14 days after pulmonary fibrosis induction significantly (*p* < 0.05) reversed the decreased lung SOD levels in comparison to BLM-G. Interestingly, no significant difference was seen in lung SOD levels between the treated groups and CN-G (Table 2, Fig. 3).

**Table 2:** Effect of BrMSCs and/or saroglitazar on lipid peroxidation parameters (MDA and SOD), the pro-inflammatory cytokines (IL-1β, IL-10, TGFß), and serum Beclin-1 in rats with BLM-induced lung fibrosis

Groups	CN-G	BLM-G	BrMSCs-G	Saro-G	BrMSCs + Saro-G
MDA (nmol/g)	36.6 ± 2.9^a^	56.3 ± 3.2^b^	51.5 ± 3.2	47.9 ± 2.6	42.2 ± 2.0^c^
SOD (u/mg)	16.7 ± 1.6^a^	13.4 ± 1.2^b^	15.7 ± 1.6^a^	16.2 ± 2.0^a^	16.5 ± 1.4^a^
IL1β (ng/ml)	59.7 ± 6.3^a^	115.5 ± 16.4^b^	100.5 ± 10.6	93.5 ± 10.6	78.0 ± 14.6^c^
IL-10 (ng/ml)	19.4 ± 2.6^a^	13.4 ± 2.0^b^	15.3 ± 1.16	15.8 ± 1.39	17.6 ± 1.57^c^
TGF-β (pg/ml)	50.5 ± 4.8^a^	155 ± 15.1^b^	128.5 ± 18.6	118.5 ± 19.7	88.5 ± 17.9^c^
Beclin-1(ng/ml)	3.0 ± 0.87^a^	1.74 ± 0.46^b^	2.35 ± 0.69	2.84 ± 0.94^c^	2.89 ± 0.62^c^

Values with the same superscript letters have no statistical significance difference

Values were expressed as (mean ± SD). One-way ANOVA was used for statistical analysis

*MDA*, malondialdehyde; *SOD*, superoxide dismutase; *IL1 β*, interleukin-1 beta; *IL-10*, interleukin-10; *TGFβ*, transforming growth factor-beta. *CN-G*, control normal group; *BLM-G*, lung fibrotic model group induced by bleomycin; *BrMSCs-G*, breast milk mesenchymal stem cells treated group; *Saro-G*, saroglitazar treated group; *BrMSCs* + *Saro-G*, breast milk mesenchymal stem cells and saroglitazar treated group

^a^(*p* < 0.05) when comparing CN-G with other groups

^b^(*p* < 0.05) when comparing BLM-G with other groups

^c^(*p* < 0.05) when comparing the BrMSCs + Saro-G group with other groups

### Effect of BrMSCs and/or saroglitazar on inflammatory mediators in the lung tissue homogenates of BLM-injected rats

The inflammatory response secondary to i.t. administration of BLM was evaluated. The protein levels of the pro-inflammatory interleukin-1-β (IL-1β) and the anti-inflammatory interleukin-10 (IL-10) were estimated. BLM administration was found to significantly (*p* < 0.05) increase IL-1β and decrease IL-10 contents in rats’ lung homogenate of BLM-G compared to CN-G. However, treatment with saroglitazar or BrMSCs initiated 14 days after pulmonary fibrosis induction significantly (*p* < 0.05) reduced the IL-1β concentration and increased IL-10 levels compared to their levels in BLM-G. Combination treatment significantly (*p* < 0.05) showed the lowest IL-1β and the highest IL-10 levels compared to other treatment groups which indicate that the optimum anti-inflammatory effect has been achieved after the concomitant administration of BrMSCs and saroglitazar (Table 2, Fig. 3).

### Effect of BrMSCs and/or saroglitazar on transforming growth factor- β (TGF- β) mRNA expression and protein levels in the lungs of BLM-injected rats

**Fig. 4:**
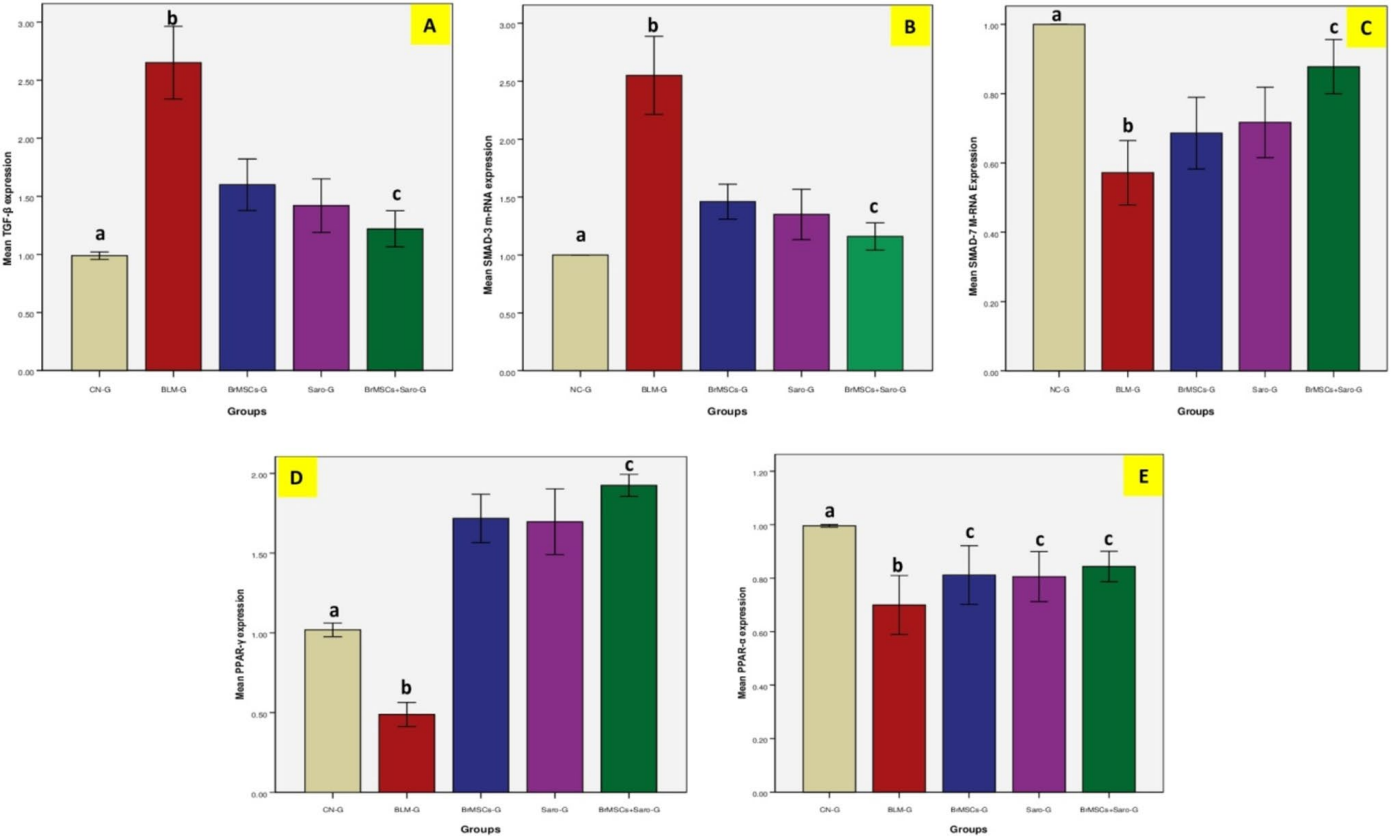
Effect of BrMSCs and/or saroglitazar on the mRNA expression genes of **A** TGF-β, **B** SMAD-3, **C** SMAD-7, **D** PPAR-γ, **E** PPAR-α in rats’ lungs with BLM-induced pulmonary fibrosis

Transforming growth factor-beta (TGF-β) in lung homogenate was detected by ELISA and real-time PCR analysis. ELISA results disclosed a significant (*p* < 0.05) high protein level after i.t. administration of bleomycin in BLM-G in comparison to CN-G. On the other hand, BrMSCs and/or saroglitazar treatments, initiated 14 days after pulmonary fibrosis induction, showed significantly (*p* < 0.05) reduced TGF-β protein levels in comparison with BLM-G. Among all treated groups the most significant (*p* < 0.05) protein values were noticed to be reduced in the combination group (BrMSCs + Saro-G) (Table 2, Fig. 3). The outcomes of real-time PCR went with the ELISA results as BLM was found to significantly (*p* < 0.05) increase TGF-β expression in BLM-G compared to NC-G. On the contrary, TGF-β expression was significantly (*p* < 0.05) decreased after treatment with BrMSCs and/or saroglitazar compared to the BLM-G. Not surprisingly, the combined treatment revealed the same best finding among the treated groups like PCR data results, as it revealed the lowest significant (*p* < 0.05) expression levels compared to the individual use of each treatment alone (Table 3, Fig. 4).

**Table 3:** Effect of BrMSCs and/or saroglitazar on transforming growth factor-beta (TGF-β), SMAD 3/7 and peroxisome proliferator-activated receptor (PPAR α/γ) expression in rats' lungs with BLM-induced pulmonary fibrosis

Groups	CN-G	BLM-G	BrMSCs-G	Saro-G	BrMSCs + Saro-G
TGF-β	1.0 ± 0.06^a^	2.65 ± 0.3^b^	1.6 ± 0.2	1.42 ± 0.23	1.22 ± 0.155^c^
SMAD-3	1 ± 0.0^a^	2.55 ± 0.33^b^	1.46 ± 0.15	1.35 ± 0.21	1.16 ± 0.11^c^
SMAD-7	1 ± 0.0^a^	0.57 ± 0.09^b^	0.68 ± 0.01	0.75 ± 0.1	0.878 ± 0.07^c^
PPAR-γ	1.0 ± 0.04^a^	0.489 ± 0.075^b^	1.69 ± 0.2	1.72 ± 0.15	1.92 ± 0.07^c^
PPAP-α	1.0 ± 0.005^a^	0.70 ± 0.11^b^	0.80 ± 0.1^c^	0.83 ± 0.08^c^	0.84 ± 0.05^c^

Values with the same superscript letters have no statistical significance difference

Values were expressed as (mean ± SD). One-way ANOVA was used for statistical analysis

*PPAR-γ*, peroxisome proliferator-activated receptor-gamma; *PPAR-α*, peroxisome proliferator-activated receptor alpha; *TGF-β*, transforming growth factor-beta; *CN-G*, control normal group; *BLM-G*, lung fibrotic model group induced by bleomycin; *BrMSCs-G*, breast milk mesenchymal stem cells treated group; *Saro-G*, saroglitazar treated group; *BrMSCs* + *Saro-G*, breast milk mesenchymal stem cells and saroglitazar treated group

^a^(*p* < 0.05) when comparing CN-G with other groups

^b^(*p* < 0.05) when comparing BLM-G with other groups

^c^(*p* < 0.05) when comparing BrMSCs + Saro-G with other groups

### Effect of BrMSCs and/or saroglitazar on SMAD 3/7 and PPAR α/γ expression in lungs of BLM-injected rats

Treatment with Br-MSCs, saroglitazar, or their combination that was initiated 14 days after the induction of pulmonary fibrosis significantly (*p* < 0.05) reduced SMAD-3 gene expression compared to the BLM-G. Combined treatment showed a more significant (*p* < 0.05) reduction in SMAD-3 gene expression as compared to individual treatments. On the other hand, values of SMAD-7 expression were significantly (*p* < 0.05) decreased in BLM-G. Nevertheless, individual, or combined treatment with BrMSCs and saroglitazar significantly (*P* < 0.05) increased SMAD-7 expression values compared to the BLM-treated group (Table 3, Fig. 4).

The relative mRNA expressions of peroxisome proliferator-activated receptors -α/γ (PPAR-α/γ) genes were significantly (*p* < 0.05) decreased in the lung tissue of BLM-G compared with CN-G. On the other hand, BrMSCs and/or saroglitazar treatment significantly (*p* < 0.05) upregulated the PPAR-α/γ expression in rats’ lung compared with BLM-G (the increase in expression values were more significantly remarkable in PPAR-γ than PPAR-α). Although, it was noted that PPAR-γ expression values were significantly (*p* < 0.05) reduced in the BrMSCs + Saro-G (combination) group, the expression values of PPAR-α in the latter group showed non-significant changes when compared with other treatment groups. Taken together, these findings suggest that BrMSCs and/or saroglitazar could inhibit BLM-activated TGF-β1/SMAD signaling pathway in pulmonary fibrosis with a remarkable role of PPARγ/α (Table 3, Fig. 4).

### Effect of BrMSCs and/or saroglitazar on autophagy markers in the lung tissue of BLM-injected rats

**Fig. 5:**
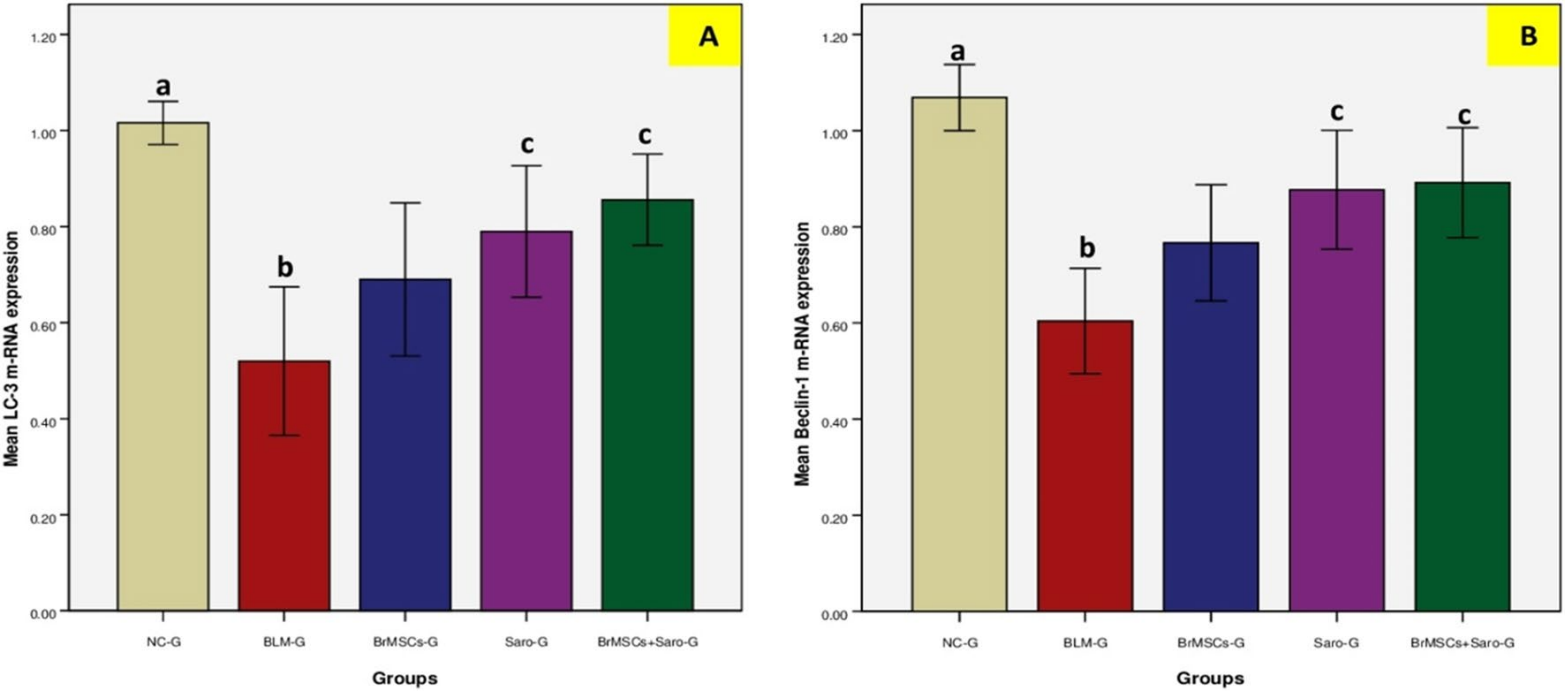
Effect BrMSCs and/or saroglitazar on mRNA levels of the autophagy markers **A** LC3 and **B** Beclin-1 in the rats’ lung tissue of BLM-induced lung fibrosis

The protein levels of the autophagy marker Beclin-1 showed at Table 2 and Fig. 3, and the mRNA levels of autophagy-related genes, LC-3 and Beclin-1, showed at Table 4 and Fig. 5 revealed significantly (*p* < 0.05) decreased values in the lung fibrosis model group (BLM-G) compared to CN-G. On the other hand, Br-MSCs and/or saroglitazar treatment, that was initiated 14 days after pulmonary fibrosis induction, showed significantly (*p* < 0.05) increased protein levels of Beclin-1 (Table 2, Fig. 3) and expression levels of LC-3 and Beclin-1 (Table 4) when compared to BLM-G. It is worth noting that the group that received a combination treatment of BrMSCs and saroglitazar showed the highest Beclin-1 protein levels (Table 2, Fig. 3) and the highest expression values of LC-3 and Beclin-1 (Table 4, Fig. 5) among all treated groups, however, this high expression was significant (*p* < 0.05) when compared to BrMSCs-G and non-significant when compared to Saro-G.

**Table 4:** Effect BrMSCs and/or saroglitazar on mRNA levels of the autophagy markers (LC3 and Beclin-1) in the rats’ lung tissue of BLM-induced lung fibrosis

Groups	CN-G	BLM-G	BrMSCs-G	Saro-G	BrMSCs + Saro-G
LC3	1.02 ± 0.05^a^	0.5 ± 0.15^b^	0.7 ± 0.16	0.79 ± 0.14^c^	0.82 ± 0.13^c^
Beclin-1	1.06 ± 0.07^a^	0.6 ± 0.11^b^	0.76 ± 0.12	0.87 ± 0.12^c^	0.89 ± 0.11^c^

Values with the same superscript letters have no statistical significance difference

Values were expressed as (mean ± SD). One-way ANOVA was used for statistical analysis

*LC3*, microtubule-associated proteins 1A/1B light chain 3B; *CN-G*, control normal group; *BLM-G*, lung fibrotic model group induced by bleomycin; *BrMSCs-G*, breast milk mesenchymal stem cells treated group; *Saro-G*, saroglitazar treated group; *BrMSCs* + *Saro-G*, breast milk mesenchymal stem cells and saroglitazar treated group

^a^(*p* < 0.05) when comparing CN-G with other groups

^b^(*p* < 0.05) when comparing BLM-G with other groups

^c^(*p* < 0.05) when comparing BrMSCs + Saro-G with other groups

### Correlation between TGF-ß & PPARα/γ, SMAD-3 & PPAR α/γ, and TGF-ß & autophagic markers

**Fig. 6:**
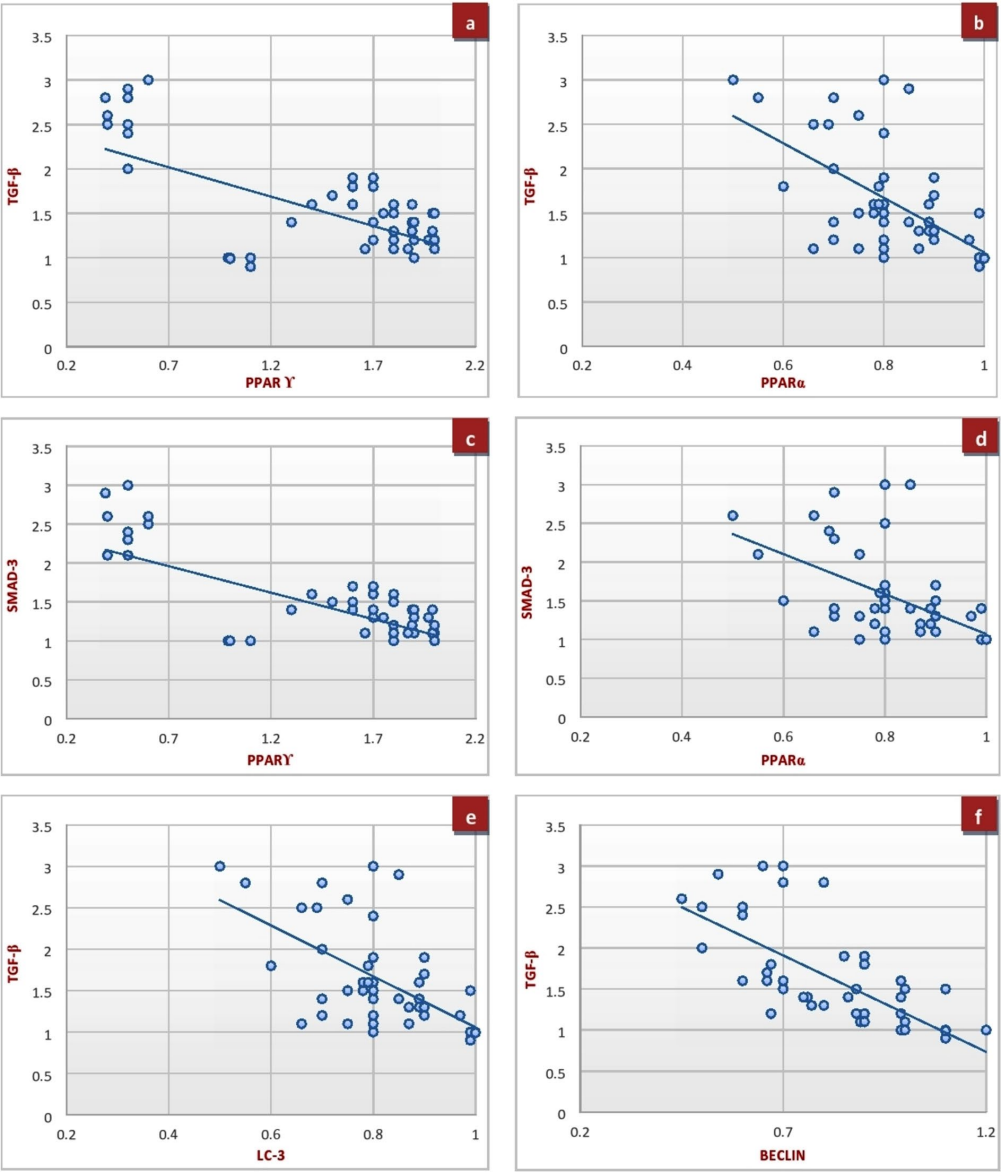
Linear relationship between TGF-ß & PPARα/γ, SMAD-3 & PPAR α/γ, and TGF-ß & autophagic markers (Pearson`s correlation**) a** significant negative correlation between TGF-β and PPAR-ϒ with correlation coefficient (r = -0.6, *P* < 0.0001), **b** significant negative correlation between TGF-β and PPAR-α with correlation coefficient (r = -0.63, *P* < 0.0001), **c** significant negative correlation between SMAD-3 and PPAR-ϒ with correlation coefficient (r = -0.65, *P* < 0.0001), **d** significant negative correlation between SMAD-3and PPAR-α with correlation coefficient (r = -0.56, *P* < 0.0001), **e** significant negative correlation between TGF-β and LC-3 with correlation coefficient (r = -0.69, *P* < 0.0001), and **b** significant negative correlation between TGF-β and Beclin with correlation coefficient (r = -0.71, *P* < 0.0001)

To determine the linear relationship between TGF-ß/SMAD-3, as the main lung fibrotic pathway regulators, with PPAR-α/γ and autophagic markers LC-3 and Beclin, Pearson’s correlation was statistically performed revealing that there was a significant negative correlation between TGF-β and PPAR-γ with correlation coefficient (r = -0.6, *P* < 0.0001). Likewise, there was a significant negative correlation between TGF-β and PPAR-α with a correlation coefficient (r = -0.63, *P* < 0.0001) (Fig. 6a, b).

Also, there was a significant (*P* < 0.0001) negative correlation between SMAD-3 and PPAR-γ with a correlation coefficient (r = -0.65) and a significant (*P* < 0.0001) negative correlation between SMAD-3and PPAR-α with a correlation coefficient (r = -0.56) (Fig. 6c, d).

Moreover, a significant (*P* < 0.0001) negative correlation was determined between TGF-ß and LC-3 at a correlation coefficient r = -0.69 (Fig. 6e). Similarly, as seen in Fig. 6f, there was a significant (*P* < 0.0001) negative correlation between TGF-β and Beclin with a correlation coefficient r = -0.71.

### Histopathological results

#### Hematoxylin and eosin stain results

**Fig. 7:**
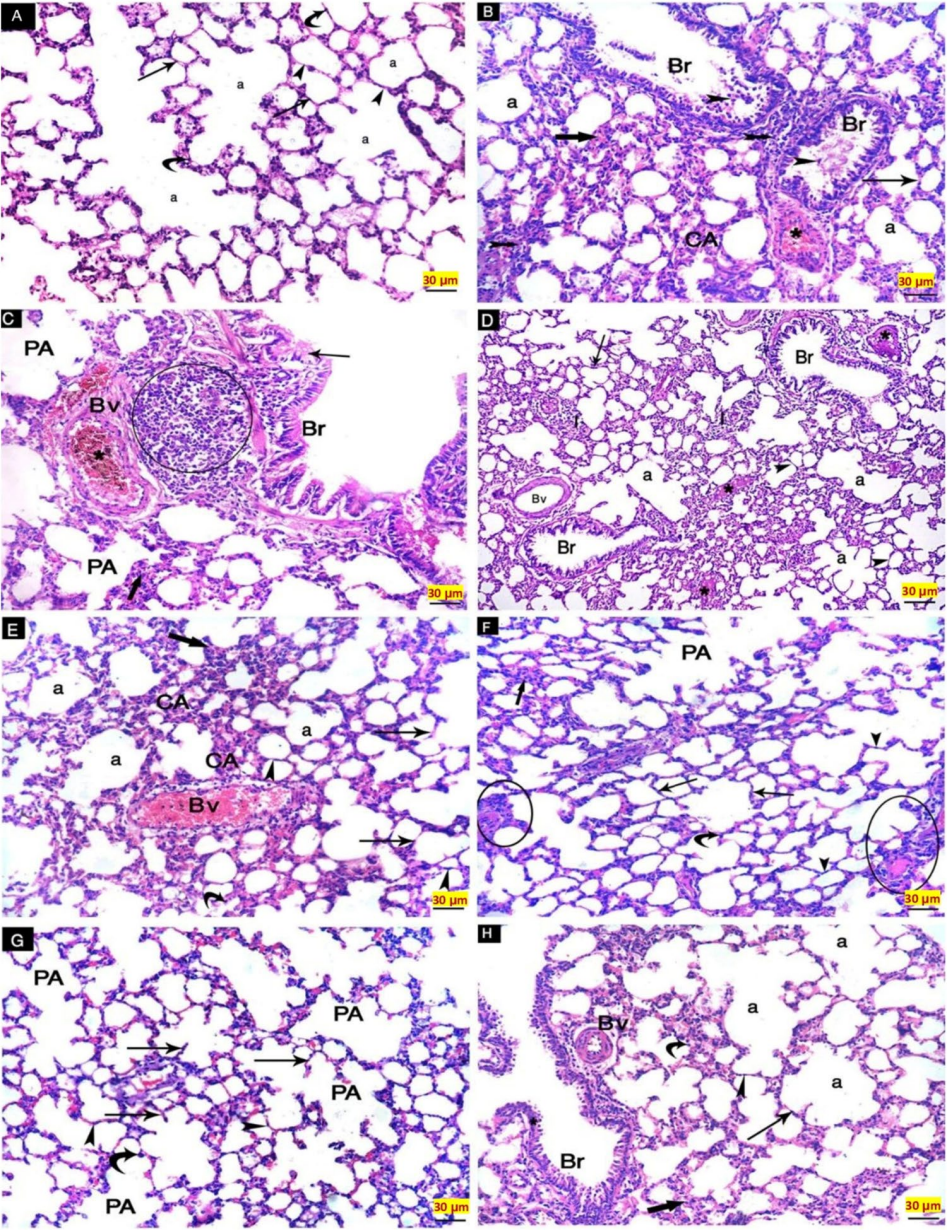
H&E-stained sections of the study groups. **A** CN-G showing normal lung architecture with rounded or polygonal alveoli (a), thin inter-alveolar septa (arrowhead). Their lining epithelium is composed of squamous type I pneumocytes with flat nuclei (thin arrow) and cuboidal type II pneumocytes with rounded nuclei (curved arrow). **B**, **C** BLM-G showing [B] Desquamated (arrowheads) bronchial (Br) epithelial cells and cellular infiltration (bifid arrows) are seen. Some patent alveoli (a) with thin inter-alveolar septa (thin arrow) and other collapsed alveoli (CA) with thick inter-alveolar septa (thick arrow). A congested blood vessel is observed (*). [C] Desquamated (thin arrow) bronchial (Br) epithelial cells and peri-bronchial cellular infiltration (circle). Some patent alveoli (PA) and other collapsed ones (thick arrow). Congested (*) blood vessel (Bv) is observed. **D**, **E** BrMSCs-G showing [D] Rounded or polygonal alveoli (a) lined with type I pneumocytes with flat nuclei (thin arrow), and thin inter-alveolar septa (arrowheads). Partially disrupted bronchial (Br) epithelium, dilated blood vessel (Bv), interstitial hemorrhage (*) and cellular infiltration (I) are still present. [E] Collapsed alveoli (CA) with thick septa (thick arrow) and patent alveoli (a) lined with type I pneumocytes with flat nuclei (arrowheads) and type II pneumocytes with rounded nuclei (curved arrow) with thin inter-alveolar septa (thin arrow). Congested blood vessel (Bv) is also present. **F** Saro-G showing patent polygonal alveoli (PA) lined with type I pneumocytes with flat nuclei (arrowheads) and type II pneumocytes with rounded nuclei (curved arrow), and thin inter-alveolar septa (thin arrows). A cellular infiltration (circles) is still present. **G**, **H** BrMSCs + Saro-G showing [G] Patent alveoli (PA) lined with pneumocyte type I with flat nuclei (arrowheads) and pneumocyte type II with rounded nuclei (curved arrow). Relatively thin septa (thin arrows) are also seen. [H] Patent alveoli (a) and alveolar sacs lined with type I pneumocytes with flat nuclei (arrowhead), and type II pneumocytes with rounded nuclei (curved arrow) with thin inter-alveolar septa (thin arrow). Intact bronchial (Br) epithelium (arrow) and blood vessel (Bv) are seen. Collapsed alveoli with thick septa (thick arrow) are still present. (H&EX200) (scale bar = 30 μm)

Control normal rats’ lungs revealed normal lung architecture with rounded or polygonal alveoli and thin inter-alveolar septa. Their lining epithelium is composed of squamous type-I pneumocytes with flat nuclei and cuboidal type-II pneumocytes with rounded nuclei (Fig. 7A). On the other hand, lung sections of BLM-G after an examination revealed that i.t. administration of bleomycin-induced massive lung fibrosis. Sections showed desquamated bronchial epithelial cells and peri-bronchial cellular infiltration. Some patent alveoli with thin inter-alveolar septa and other collapsed alveoli with thick inter-alveolar septa. A congested blood vessel is observed (Fig. 7B, C).

Therapies started 14 days after the induction of pulmonary fibrosis reveled that, treatment with BrMSCs demonstrated an improvement in rats’ lung histopathological sections. Animals’ lung sections of BrMSCs-G showed rounded or polygonal patent alveoli lined with type I pneumocytes with flat nuclei and thin inter-alveolar septa. Partially disrupted bronchial epithelium and dilated blood vessels were noticed. Interstitial hemorrhage and cellular infiltration are still present. The alveoli collapsed, and the septa were thick (Fig. 7D, E). In the same context, examination of saroglitazar treated group (Saro-G) lung sections revealed patent lung alveoli lined with type-I pneumocytes with flat nuclei and type-II pneumocytes with rounded nuclei, thin inter-alveolar septa, and cellular infiltration were still present (Fig. 7F). The lung sections in the combination group received BrMSCs and saroglitazar concomitantly revealed patent alveoli lined with pneumocyte type-I with flat nuclei and pneumocyte type II with rounded nuclei. Relatively thin septa are also seen. Intact bronchial epithelium and blood vessels are seen. Collapsed alveoli with thick septa were still present (Fig. 7G, H).

#### Mallory’s trichrome stain results

**Fig. 8:**
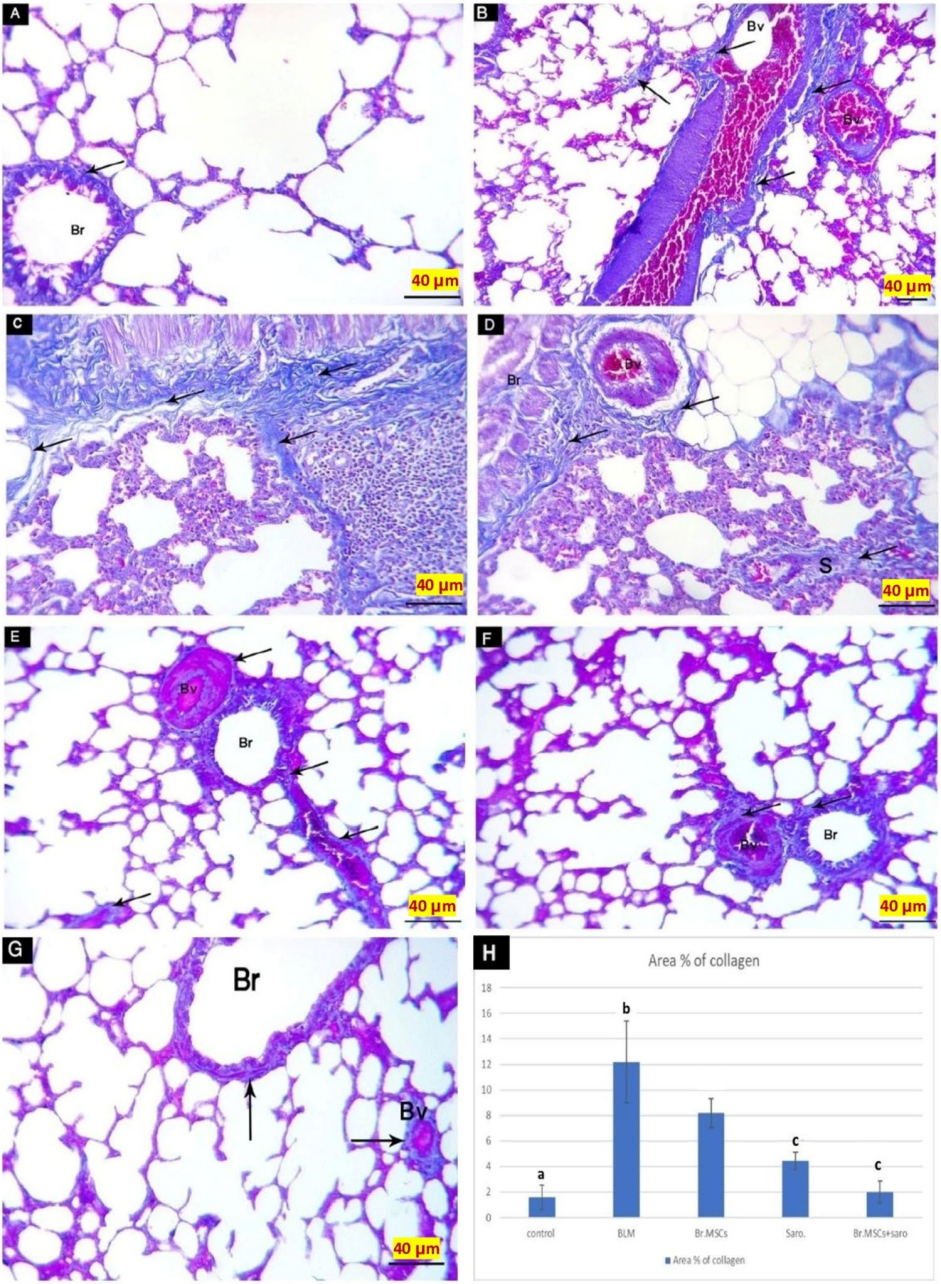
Mallory trichrome stained sections of the study groups **A** CN-G showing scanty collagen fibers (arrow) appear in the bronchial (Br) wall. **B-D** BLM-G sections showing [B] Marked deposition of collagen fibers (arrows) in the septa (S) and around blood vessels (Bv) which appear with marked congestion. **C** Marked deposition of collagen fibers (arrows) appears in the bronchial wall. [D] Marked deposition of collagen fibers (arrows) in the septa (S), around blood vessels (Bv), and in the bronchial (Br) wall. **E** BrMSCs-G sections showing [E] Few amounts of collagen fibers (arrows) in the wall of blood vessels (Bv) and the bronchial (Br) wall. **F** Saro-G showing minimal amount of collagen fibers (arrows) in the wall of blood vessels (Bv) and in the bronchial (Br) wall. **G** BrMSCs- + Saro-G showing scanty collagen fibers (arrows) appear in the bronchial (Br) wall and the wall of the blood vessel (Bv). (Mallory trichrome X400) (scale bar = 40 μm). **H** Mean values of % area of collagen among different studied groups, one way ANOVA was used for statistical analysis. “a” means value is statistical significance when comparing CN-G with other groups, “b” means the value is statistical significance when comparing BLM-G with other groups, “c” means value is statistical significance when comparing BrMSCs + Saro-G with other groups. The same letters mean no statistical significance difference

Rats’ lungs of the studied groups revealed that CN-G sections had scanty collagen fibers appearing in the bronchial wall (Fig. 8A). Bleomycin-induced lung fibrosis group (BLM-G) showed marked deposition of collagen fibers in the septa, bronchial wall and around blood vessels which appear with marked congestion (Fig. 8B-D). Bleomycin-induced lung fibrosis treated with breast milk stem cells (BrMSCs-G) had a few amounts of collagen fibers in the wall of blood vessels and the bronchial wall (Fig. 8E). Bleomycin-induced lung fibrosis treated with saroglitazar (Saro-G) had a minimal amount of collagen fibers in the wall of blood vessels and in the bronchial wall (Fig. 8F). Bleomycin-induced lung fibrosis treated with both saroglitazar and breast milk stem cells (BrMSCs + Saro-G) had relatively few collagen fibers (arrows) appear in the bronchial wall and the wall of the blood vessel (Fig. 8G).

#### Immunohistochemical stain results

**Fig. 9:**
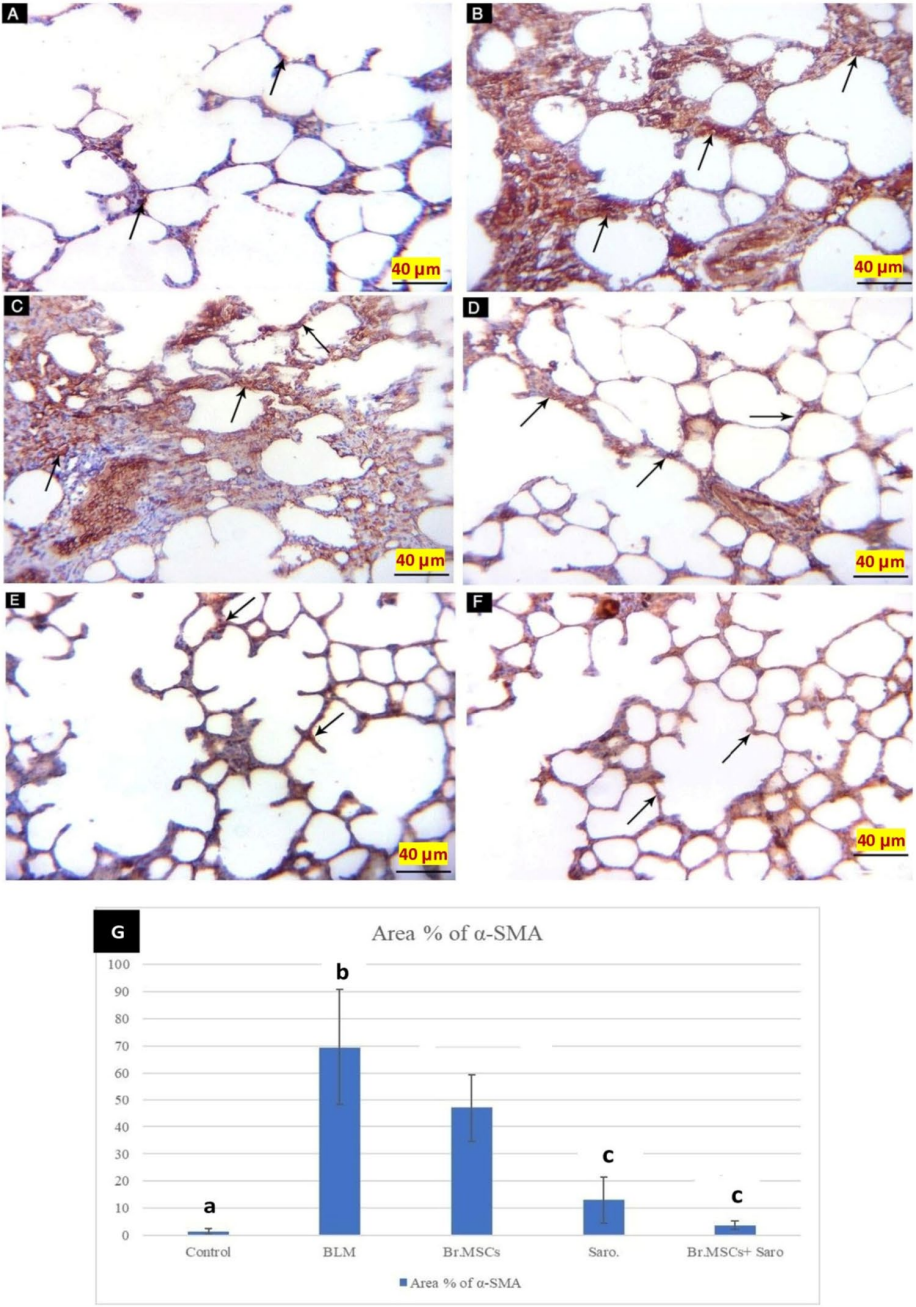
A photomicrograph of immune-stained sections of the study groups. **A** CN-G showing minimal positive immune reaction for α-SMA (arrows) at smooth muscle knobs. **B**, **C** BLM-G sections showing [B] marked α-SMA positive immune reaction (arrows) within the inter- alveolar septum. **C** Marked α-SMA positive immune reaction (arrows) within the inter-alveolar septum. **D** BrMSCs-G showing moderate positive immune reaction for α-SMA (arrows) within the inter-alveolar septum. **E** Saro-G showing mild positive immune reaction for α-SMA (arrows) within the inter-alveolar septum. **F** BrMSCs + Saro-G showing mild positive immune reaction for α-SMA (arrows) within the inter-alveolar septum and at knobs. (α-SMA X 400) (scale bar = 40 μm). **G** Mean values of % area of α-SMA among different studied groups, one way ANOVA was used for statistical analysis. “a” means value is statistical significance when comparing CN-G with other groups, “b” means the value is statistical significance when comparing BLM-G with other groups, “c” means the value is statistical significance when comparing BrMSCs + Saro-G with other groups. The same letters mean no statistical significance difference

Immuno-stained sections of rats’ lungs with anti- αSMA of study groups revealed that CN-G showed minimal positive immune reaction for α-SMA at smooth muscles knobs (Fig. 9A). Bleomycin-induced lung fibrosis group (BLM-G) had marked α-SMA positive immune reaction (Fig. 9B, C). BrMSCs-G pulmonary sections showed moderate positive immune reaction for α-SMA (Fig. 9D). Sarorglitazar treated group pulmonary sections (Saro-G) had mild positive immune reaction for α-SMA (Fig. 9E). Sections of the BrMSCs + Saro-G group had mild positive immune reaction for α-SMA (Fig. 9F).

**Fig. 10:**
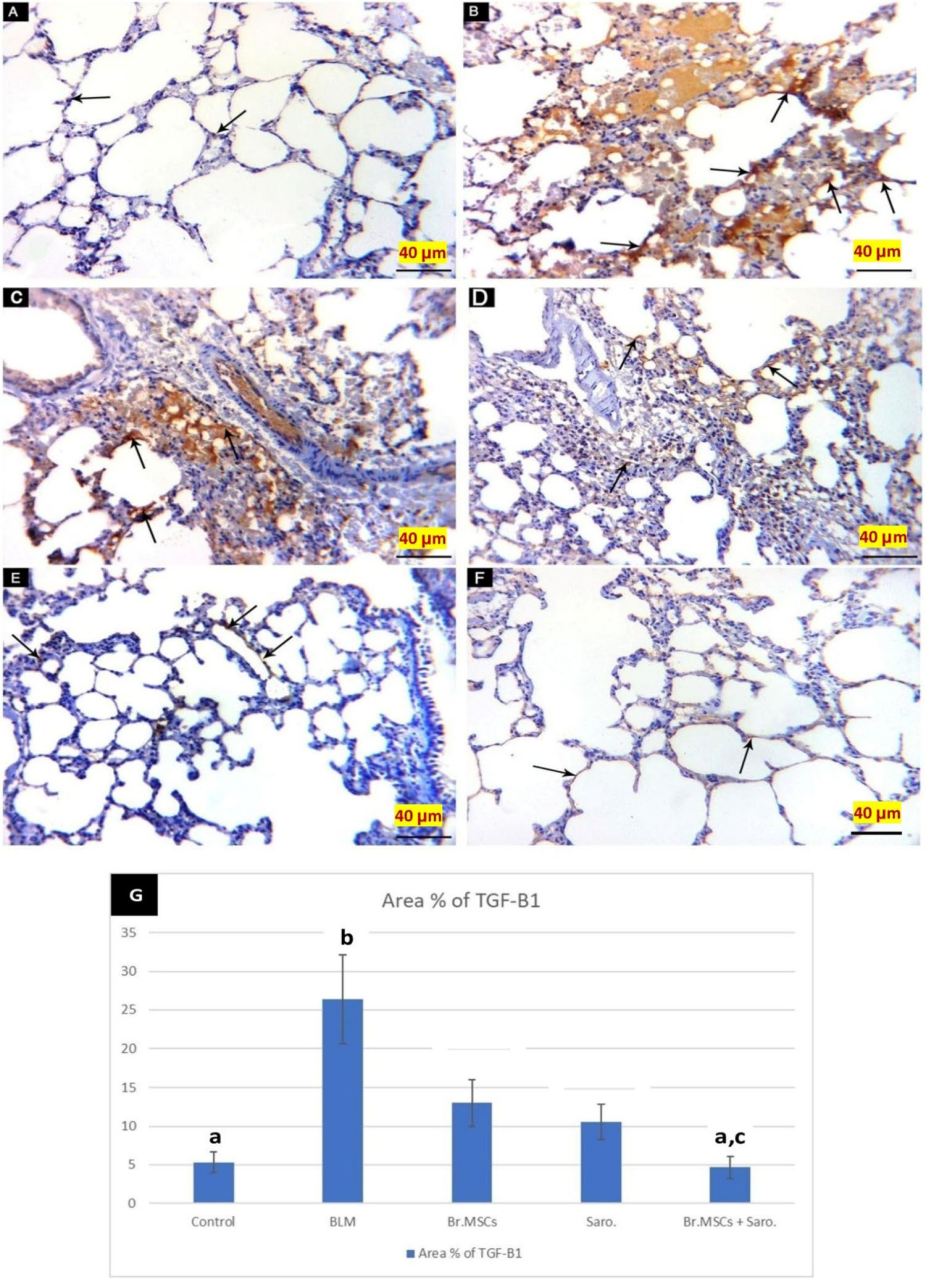
A photomicrograph of immune-stained sections of the study groups. **A** CN-G showing faint expression of TGF-β1 by some cells in the alveolar septa between the alveoli and alveolar sacs. **B**, **C** BLM-G sections showing [B] Numerous cytoplasmic reactions for TGF-β1 positive immune expressed cells (arrows) within the inter-alveolar septum. **C** Increased TGF-β1 positive immune expressed cells (arrows) within the inter-alveolar septum. **D** BrMSCs-G showing some positive immune expressed cells (arrows) for TGF-β1 within the inter-alveolar septum. **E** Saro-G showing few positive immune expressed cells (arrows) for TGF-β1 within the inter-alveolar septum. **F** BrMSCs + Saro-G showing faint weak positive TGF-β1 immune expressed cells (arrows) within the inter-alveolar septum. (TGF-β1 X 400) (scale bar = 40 μm). **G** Mean values of % area of TGF-ß1 among different studied groups, one way ANOVA was used for statistical analysis. “**a**” means value is statistical significance when comparing CN-G with other groups, “**b**” means the value is statistical significance when comparing BLM-G with other groups, “**c**” means the value is statistical significance when comparing BrMSCs + Saro-G with other groups. The same letters mean no statistical significance difference

Immuno-stained sections of rats’ lungs with anti-TGF-β1 of study groups revealed that the control group showed faint expression of TGF-β1 by some cells in the alveolar septa between the alveoli and alveolar sacs (Fig. 10A). Bleomycin-induced lung fibrosis group (BLM-G) had numerous cytoplasmic reactions for TGF-β1 positive immune expressed cells within the inter-alveolar septum (Fig. 10B, C). BrMSCs-G had some positive immune expressed cells for TGF-β1 within the inter-alveolar septum (Fig. 10D). Saro-G showed few positive immunes expressed cells for TGF-β1 within the inter-alveolar septum (Fig. 10E). BrMSCs + Saro-G group revealed faint weak positive TGF-β1 immune expressed cells within the inter-alveolar septum (Fig. 10F).

#### Morphometric and statistical results

Regarding the mean ± SD values for the area % of collagen fibers, there was a highly statistically significant difference between BLM-G as compared to CN-G (the least values in the CN-G and highest values in the BLM-G). On the other hand, in BrMSCs-G, Saro-G, and Br-MSCs + Saro-G the area % of collagen was significantly reduced in comparison to BLM-G. While comparing the treated groups, MSCs + Saro-G showed the most significant reduction in the area % of collagen fibers (Fig. 8).

Concerning the mean ± SD values for the area % α-SMA immune reaction, there was a statistically significant difference between all studied groups as the least significant levels were observed in the CN-G while the highest levels were noticed in the BLM-G. Conversely, the area of α-SMA immune reaction in all treated groups was statistically significantly lower than the BLM-G with the least significant levels observed in the BrMSCs + Saro-G (Fig. 9).

Regarding the mean ± SD values for the area % TGF-β1 immune reaction, there were statistically significant high values in BLM-G when compared to CN-G. However, treatment with Br-MSCs and /or saroglitazar revealed significantly low values in comparison to BLM-G values. In comparison between all treated groups, BrMSCs + Saro-G showed the least significant values of TGF-β1 immune reaction area (Fig. 10).

## Discussion

Lung fibrosis persists to be an enduring medical challenge due to the limited availability of effective therapies. Hence, the demand to search for different treatment options is continuous. The present work was conducted to investigate the potential roles of BrMSCs and saroglitazar in alleviating pulmonary fibrosis (PF) and to compare the results of every single therapy with the concomitant use of both treatments. The study also shed light on the mechanisms by which the used therapies mitigated PF.

In the current study, PF was induced by intratracheal (i.t.) injection of BLM. Based on previous research productions, it is the most preferred model for experimental works. This could be justified by the direct toxic effects of BLM on the lung tissue after its i.t instillation that generates a typical classical picture of PF (Zakaria et al. 2021; Egger et al. 2013). The outcome of our present study revealed that BLM caused devastating pulmonary effects. The scarce lung production of the hydrolase enzyme makes the lungs more vulnerable to BLM toxic effects (Turgut et al. 2016). Conversely, breast milk mesenchymal stem cells (BrMSCs) and saroglitazar therapies, that were initiated 14 days post PF induction, restored the oxidative/antioxidant imbalance initiated by BLM. In the meantime, the pulmonary pro-inflammatory interleukin-1ß (IL-1ß) and the pro-fibrotic transforming growth factor beta (TGF-ß) cytokines were decreased; however, the production of the anti-inflammatory interleukin-10 (IL-10) was increased. Also, BrMSCs and saroglitazar down-regulated SMAD-3 however; SMAD-7 was upregulated. Furthermore, the autophagy-related genes, LC3 and Beclin, were downregulated after treatment, and the expression of peroxisome proliferator activated receptor α/γ (PPAR-α and γ) was increased. At the level of histological and immunohistochemical studies, the pulmonary tissue derangements and fibrosis seen by i.t. BLM injection were ameliorated.

Body weight (BW) is one of the well-known toxicity symptoms of BLM (Attia et al. 2017). In the current study, BLM significantly led to rats’ BW loss with a significant increase in lung index; these outcomes are similar to Turgut and coworkers findings (2016). Cowley et al. attributed that to the wasting of rats’ skeletal muscle and fat after exposure to BLM and further explained that by the diminished eating behavior of animals which in turn affects fat stores for energy production. On the other hand, the increase in lung index was attributed not only to the BW reduction but also to the gain in lung weight due to fluid accumulation, increased inflammation, and fibrosis secondary to BLM instillation (Cowley et al. 2019).

Impressively, BrMSCs treatment significantly halted the decreased BW that resulted from i.t. BLM injection which agrees with our previous work (Nageeb et al. 2022). Furthermore, lung index was reduced after BrMSCs treatment. The restoration of lung weight to normal and the increase in rats’ BW are believed to be the cause. This was further supported by the improved lung histopathological picture that was positively reflected on lung weight leading to fluid accumulation reduction and tapering off inflammation and fibrosis. In the same context and agreement with Hassan et al. (2019), saroglitazar was found to normalize rats’ BW in the current study. The recovery of lung histological structure with marked abatement of inflammation, tissue edema, and fibrosis were the principal reasons for the decreased lung index in saroglitazar-treated rats.

Cytotoxicity of BLM encompasses redox imbalance as one of the induced lung injury mechanisms (Iyer et al. 2009). Consistent with Zakaria et al. (2021) we found that BLM incentivized this redox imbalance evidenced by the increased levels of the oxidative marker malondialdehyde (MDA) and the reduced antioxidant superoxide dismutase (SOD) activities. Allawzi and co-workers (2019) explained that BLM produces reactive oxygen species (ROS) after making a complex in tissues with ferrous iron (Fe2 +) and subsequently oxidizing to ferric iron (Fe3 +) leading to the reduction of oxygen to free radicals. Thus, the formed free radicals attack cell membranes causing lipid peroxidation. Additionally, DNA damage occurs after the binding of the BLM complex with a nucleophilic bond to the DNA helix.

Further evidence of pulmonary injury driven by BLM in our study was reported by the markedly distorted picture of lung architecture as seen in the BLM-G-stained H&E lung sections in the form of desquamated bronchial epithelial cells and peri-bronchial cellular infiltration. Some patent alveoli with thin inter-alveolar septa and other collapsed alveoli with thick inter-alveolar septa and congested blood vessels were also observed. The aforementioned findings came in line with many studies findings (Zakaria et al. 2021; Li et al. 2021). Likewise to our current work finding, several studies reported that BLM intake negatively affects most of the lung tissues in the form of bronchial epithelial cells desquamation and peri-bronchial cellular infiltration, alveolar collapse and marked thickening of the inter-alveolar septa as well as severely congested blood vessels (Zakaria et al. 2021; Li et al. 2017). The presence of additional inflammatory cells, extravasated RBCs, and clogged capillaries may be regarded as the cause of the apparent thickening of the inter-alveolar septa. The activation of macrophages, which produce interleukin-8 (a potent neutrophil chemotactic), and other inflammatory cells was the cause of the increase in inflammatory cellular infiltration in the lung. They become more prevalent in the vascular spaces and interstitium (Hickey et al. 2016). Additionally, Mallory trichrome stain further proved the induced PF by the obvious statistically confirmed increase in the pulmonary collagen deposition compared to CN-G which is following the finding of Liu and co-authors (Liu et al. 2017a, b). Reinert et al. suggested that stimulation of the alveolar macrophages by BLM leads to the production of inflammatory and profibrotic cytokines (Reinert et al. 2013). Additionally, hyperplasia of type-II pneumocytes secondary to tracheal BLM instillation leads to inflammatory and fibrotic cytokines secretion (Kim et al. 2019). As a result, some of these cytokines cause proliferation and activation of fibroblasts and transformation to myofibroblasts leading to increased deposition of collagen ending by the collapse of alveoli; however, others cause inflammatory cell recruitment and cellular infiltration by their chemoattractants behavior (Dong et al. 2017). Furthermore, the high permeability of pulmonary blood capillaries assists in the inflammatory cell infiltration and could also elucidate the observed vascular congestion in lung sections of BLM-G. The aforementioned explanations not only clarify the histological findings of our work but also interpret the significant increase in the pro-inflammatory IL-1 and the pro-fibrotic TGF-ß markers and the decrease in the anti-inflammatory cytokine IL-10 in the current work. Not impressively, Liu et al. (2017a, b) reported the same increase in the inflammatory and fibrotic cytokines after induction of experimental PF by BLM.

Although the use of mesenchymal stem cells (MSCs) in PF is not investigated yet, their effectiveness in different lung lesions has been widely reported in literatures which attributed that to the pleiotropic (immunomodulatory, anti-inflammatory, and antifibrotic) behavior of MSCs (Xu et al. 2018; Cruz and Rocco 2020; Nasri et al. 2021). Li and colleagues affirmed that the injured lungs release many chemotactic mediators which interact and adhere with their surface ligands on MSCs (Li et al. 2018). Among those mediators, the hepatocyte growth factor (HGF) is considered the most efficient factor for MSCs migration. In the current study, BrMSCs proved their homing ability to the site of lung injury following their intraperitoneal administration. Consistent with Hu and coworkers (Hu et al. 2022) our data showed that BrMSCs therapy significantly ameliorated the oxidative, inflammatory, and fibrotic responses caused by BLM as well as improved lung autophagy and the histological picture. Research declared that MSCs can promote macrophage polarization from pro-inflammatory to anti-inflammatory state through the production of immunosuppressive factors. Meanwhile, MSCs could secret fibrosis degrading enzymes as matrix metalloproteinase (Hardjo et al. 2009). Meanwhile, MSCs can inhibit the B-cells maturation and recruitment in the damaged lung during IPF; therefore, the inflammatory and fibrotic process is inhibited (Kletukhina et al. 2022). Furthermore, MSCs can reduce T cell immune responses and promote immune regulatory cytokines generation (Cargnoni et al. 2020). Interestingly, in the current study, Mallory trichrome stained sections of BrMSCs-G had few amounts of collagen fibers in the wall of blood vessels and in the bronchial wall which agrees with Mahmoudi et al. findings’ (2020). The MSCs’ efficacy in PF is also linked to their ability to diminish the deposition of collagen and secrete anti-inflammatory and anti-fibrotic elements that in turn promote lung tissue repair (Shi et al. 2021). Moreover, MSCs secrete prostaglandin E2 (PGE2) and hepatocyte growth factor (HGF) which reduces the major factors that enhance ECM deposition in type-II alveolar cells (Zanoni et al. 2019). Additionally, HGF prevents lung tissue thickening via activating matrix metalloproteinase 1 (MMP-1) which acts as collagenase. Conclusively, MSCs regenerative power relies on their ability to restore the structure and function of injured lung via their paracrine effects (Chen et al. 2022).

BLM instillation significantly decreased Beclin-1 and LC3-II, in the present work which agrees with El-Horany et al., work (2023). Interestingly, enhancement of autophagy was reported in various lung diseases like asthma (Poon et al. 2017) and chronic obstructive pulmonary disease (Tan et al. 2019) leading to disease progression. On the other hand, controversial observations were reported when talking about autophagy and PF (Hill and Wang 2022). Despite the activation of pathways that promote autophagy, Patel and colleagues (2012) have demonstrated that autophagy is not induced in PF attributing that to the high TGF-ß1 levels. They suggested that TGF-β1 suppresses the autophagy via SMAD-dependant or non-dependant pathways which comply with our study outcomes. In contrast to our findings, TGF-β1 was found to stimulate autophagy in other studies (Xia et al. 2020; Kaushal et al. 2020; Orogo and Gustafsson 2015). This controversial autophagy behavior in fibrogenesis could be in part attributed to the different organs types. In the same context, we statistically confirmed that BLM increased α SMA expression in the immunohistochemical sections which correspond with Liu and colleague’s work (2022). Being the most accepted indicator for PF, α-SMA low expression results in reduction in extracellular matrix (ECM) deposition which directly reflected in the alveolar wall thickening and ventilation function (Liu et al. 2022). Patel et al. (2012) suggested that suppression of autophagy is the cause, as autophagy serves as a degradation pathway for intracellular proteins, and upon its inhibition collagen degradation is inhibited leading to its accumulation.

BrMSCs therapy enhanced autophagy in our current study which agrees with other works findings (Chen et al. 2020a, b (A); Chen et al. 2020a, b (B)). In contrast, Zhu and coworkers (2016) concluded that MSCs inhibits alveolar macrophage autophagy in PF. Another finding summarized that bone marrow-derived MSCs mitigate PF via inhibiting apoptosis and pyroptosis but not autophagy (Zhao et al. 2021). These contentious autophagy effects by MSCs might be due to targeting different biological signals or pathways.

TGF-β, the master regulatory factor in PF, enhances fibroblasts and myofibroblasts activation directly via SMAD dependent pathway (Wei et al. 2021). We assessed TGF-β in this present work by different ways to prove its pivotal role in fibrogenesis process and its dependency on the SMAD proteins. We statistically confirmed that the immune-stained sections of BLM-G have numerous positive cytoplasmic reactions for TGF-β1 within the inter-alveolar septum as compared to CN-G which is in line with the results of previous studies (Attia et al. 2017; Gad et al. 2020). Notably, TGF-β modulates pro-fibrotic genes via activation of cytoplasmic SMAD-2 and SMAD-3 which in turn the induction of lung epithelium-mesenchymal transition (EMT) takes place and as a result, the propagation of fibrogenic process is augmented (Gad et al. 2020). Conversely, the activated TGF-β diminishes the expression of the antifibrotic regulatory protein, SMAD-7 which matches our study results (Xu et al. 2009). On the other hand, the homed BrMSCs in lung tissues of the present study inhibited TGF-β and SMAD-3 however; SMAD-7 expression was enhanced. Interestingly, several studies reposted similar findings confirming that the exerted robust anti-inflammatory/anti-fibrotic effects of MSCs are done via targeting the canonical SMAD pathway (Wei et al. 2021; Gazdhar et al. 2013).

Saroglitazar, a full agonist of PPAR-α/γ, shows multiple pleiotropic effects on different organs mainly affecting various metabolic pathways. So far, the anti-fibrotic role of saroglitazar in organ fibrosis is promising especially in hepatic fibrosis; however, its role in PF is not investigated yet (Yoon et al. 2015) Thus, we have raised an important question regarding the role of saroglitazar in PF, and its role in hastening the resolution of pulmonary inflammation and fibrosis either when used individually or concomitantly with BrMSCs. The answer came quickly as saroglitazar significantly restored the oxidative/antioxidant balance, diminished the inflammatory response, and reduced the fibrotic markers while enhancing the anti-fibrotic and autophagic markers. Moreover, the H&E and Mallory trichrome and immunohistochemical stained lung sections were greatly improved especially when added to the BrMSCs and all these findings were statistically confirmed. Several experimental studies revealed the impressive anti-inflammatory/ antifibrotic effects of saroglitazar with its ability to reverse fibrosis (Khamis et al. 2021; Jain et al. 2018).

Remarkably, studies reported the vital role of PPAR agonists in fibrogenesis through inhibiting TGF-ß-driven differentiation of myofibroblast and the production of type-I collagen protein (Burgess et al. 2005; Tan et al. 2010). In PF murine model, PPARγ agonists induced cell cycle arrest and inhibited the lung proliferative responses induced by bleomycin (Milam et al. 2008). Also, PPAR-α activation is essential for lung function recovery and cessation of the fibrotic process (Liu et al. 2017a, b). Furthermore, PPAR-α expression was noticed to inversely correlate with TGF-β1 expression. In the same context, the pan PPAR agonist IVA337 revealed a marked reduction in the activated TGF-β in PF (Avouac et al. 2017). The preceding works have demonstrated the roles of PPARs activation in attenuating the lung fibrogenic process which is consistent with our current findings that revealed the significance of PPAR α and γ in ameliorating PF and how their expressions were negatively correlated to TGF-β1 and SMAD-3. Moreover, the negative correlation between TGFß and autophagy markers was confirmed denoting the influential role of autophagy in abating TGFß, the displeasing fibrogenic cytokine. These data were directly reflected in the improved lung histology in saroglitazar treated rats.

Our results implied that PF was attenuated by targeting the TGF-β/SMAD pathway partially through activating PPAR α/γ. This could be a logically accepted protective mechanism exerted by saroglitazar but impressively it is an additive mechanism of protection wielded by BrMSCs against PF. Based on that not surprisingly rats received both saroglitazar and Br-MSCs showed the top results compared to those received each treatment alone.

## Conclusion

In conclusion, our present work presented loads of evidence that suggest that interrupting the TGFß/SMAD pathway by BrMSCs and/or saroglitazar could impair the vicious circle of lung fibrosis induced by BLM. We confirmed that BrMSCs and/or saroglitazar can exert antioxidant, anti-inflammatory, and anti-fibrotic roles and enhance lung tissue autophagy in PF. Rats who received combined therapy experienced better therapeutic effects than those who received individual therapy. Our study also determined that PPAR α/γ activation might in part ameliorate lung tissue fibrosis, which shed light on the promising roles of the dual PPAR agonist therapies on PF.

Although this study contributed to the understanding of the main mechanisms of BrMSCs and saroglitazar in protecting against IPF, further studies are still needed to reveal the specific molecular and cellular targets of each therapy.

### Supplementary Information

Below is the link to the electronic supplementary material.

Supplementary file1 (DOCX 15 KB)

## Data Availability

No datasets were generated or analysed during the current study.
