# Supplementary material for: Alleviation of pulmonary fibrosis by the dual PPAR agonist saroglitazar and breast milk mesenchymal stem cells via modulating TGFß/SMAD pathway
Source: Naunyn Schmiedebergs Arch Pharmacol. 2024 Feb 20;397(8):5953–74. doi: 10.1007/s00210-024-03004-y (PMC11329427; doi:10.1007/s00210-024-03004-y)
Supplement: Supplementary file 1 — Supplementary file1 (DOCX 15 KB) [file 210_2024_3004_MOESM1_ESM.docx]

**Alleviation of pulmonary fibrosis by saroglitazar and Breast milk mesenchymal stem cells via modulating TGFß/SMAD pathway**

**Seba Hassan Attia^1*^, Sara F. Saadawy^2^, Samaa M. El-Mahroky^3^, Mahitab M. Nageeb^1^**

**^1^** Clinical Pharmacology Department, Faculty of Medicine, Zagazig University, Zagazig, Egypt.

**^2^** Medical Biochemistry and Molecular Biology Department, Faculty of Medicine, Zagazig University, Zagazig, Egypt.

**^3^** Medical Histology and Cell Biology Department, Faculty of Medicine, Zagazig University, Zagazig, Egypt.

Authors details:

***Seba Hassan Attia (Corresponding author);** Lecturer of Clinical Pharmacology, Faculty of Medicine, Zagazig University, Zagazig, Egypt, e-mail: [SHAttia@medicine.zu.edu.eg](mailto:SHAttia@medicine.zu.edu.eg) **ORCID**: 0000-0001-7105-168X

**Sara F Saadawy**; Lecturer of Medical Biochemistry and Molecular Biology, Faculty of Medicine, Zagazig University, Egypt; e-mail: [SFSaadawi@medicine.zu.edu.eg](mailto:SFSaadawi@medicine.zu.edu.eg).

**Samaa M. El-Mahroky**; Lecturer of Medical Histology and Cell Biology, Faculty of Medicine, Zagazig University, Zagazig, Egypt; e-mail: [SMMahrouqy@medicine.zu.edu](mailto:SMMahrouqy@medicine.zu.edu).eg [-drnoorsamaa@gmail.com](mailto:-drnoorsamaa@gmail.com).

**Mahitab M. Nageeb**; Lecturer of Clinical Pharmacology, Faculty of Medicine, Zagazig University, Egypt; e-mail [MMAbdulWahab@medicine.zu.edu.eg](mailto:MMAbdulWahab@medicine.zu.edu.eg).

**Supplementary table (S1): Primer sequences used for quantitative real-time PCR assays**

| **Gene** | **Forward primer (5′–3′)** | **Reverse primer (5′–3′)** | **Accession No** | **Product size** |
| --- | --- | --- | --- | --- |
| Rat Gapdh | GCA TCT TCT TGT GCA GTG CC | TAC GGC CAA ATC CGT TCA CA | NM_017008.4 | 74 |
| PPAR-ϒ | GAG ATC CTC CTG TTG ACC CAG | CCA CAG AGC TGA TTC CGA AGT | NM_013124.3 | 129 |
| PPAR-α | GTCCTCTGGTTGTCCCCTTG | GTCAGTTCACAGGGAAGGCA | NM_013196.2 | 176 |
| Smad-3 | CTG GGC AAG TTC TCC AGA GTT | AAG GGC AGG ATG GAC GAC AT | NM_013095.3 | 148 |
| Smad-7 | GAG TCT CGG AGG AAG AGG CT | CTG CTC GCA TAA GCT GCT GG | NM_030858.2 | 84 |
| TGF-β1 | AGG GCT ACC ATG CCA ACT TC | CCA CGT AGT AGA CGA TGG GC | NM_021578.2 | 168 |
| Beclin-1 | GAA TGG AGG GGT CTA AGG CG | CTT CCT CCT GGC TCT CTC T | NM_001034117.1 | 180 |
| LC-3 | GAA ATG GTC ACC CCA CGA GT | ACA CAG TTT TCC CAT GCC CA | NM_012823.2 | 147 |
| Human Gapdh | GGAGTCAACGGATTTGGTCGT | ACGGTGCCATGGAATTTGC | NM_002046.7 | 161 |
| Human CD9 | TCGCCATTGAAATAGCTGCGGC | CGCATAGTGGATGGCTTTCAGC | NM_001769 | 149 |
| Human  CD11b | GGAACGCCATTGTCTGCTTTCG | ATGCTGAGGTCATCCTGGCAGA | NM_000632 | 131 |
